# 5-(Dimethyl­ammonio)naphthalene-1-sulfonate dihydrate

**DOI:** 10.1107/S1600536809044596

**Published:** 2009-11-07

**Authors:** Zuo-an Xiao, Dan Zhan

**Affiliations:** a School of Chemical Engineering and Food Science, Xiangfan University, Xiangfan 441053, People’s Republic of China

## Abstract

There are two formula units in the asymmetric unit of the title compound, C_12_H_13_NO_3_S·2H_2_O. In the crystal structure, mol­ecules are linked by inter­molecular O—H⋯O, N—H⋯O and weak C—H⋯O hydrogen bonds, forming a three-dimensional network.

## Related literature

For potential applications of the title compound, see: Chimiak & Polonski (1973 ▶).
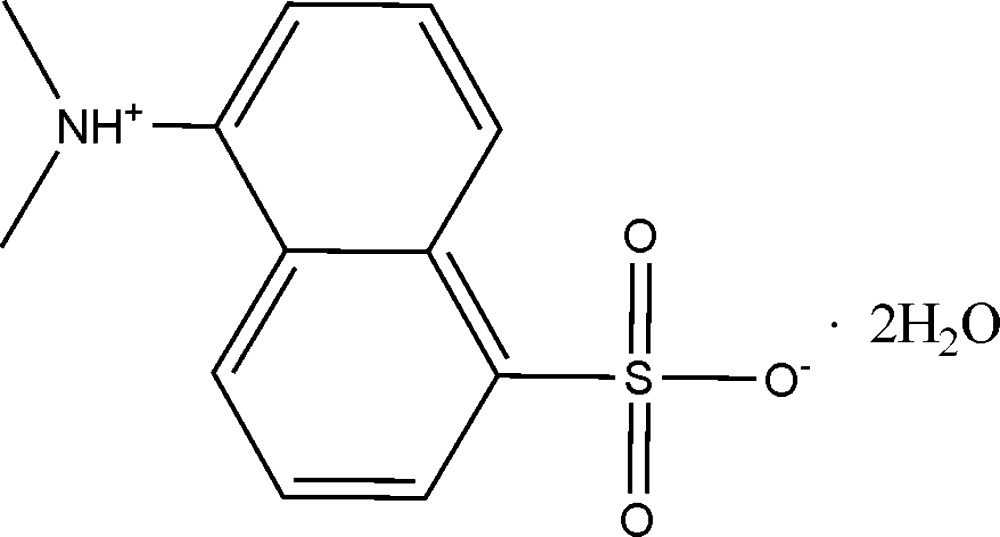


## Experimental

### 

#### Crystal data

C_12_H_13_NO_3_S·2H_2_O
*M* *_r_* = 287.33
Monoclinic, 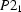

*a* = 8.1179 (7) Å
*b* = 7.7383 (7) Å
*c* = 21.4249 (19) Å
β = 91.527 (1)°
*V* = 1345.4 (2) Å^3^
*Z* = 4
Mo *K*α radiation
μ = 0.26 mm^−1^
*T* = 298 K
0.23 × 0.10 × 0.10 mm

#### Data collection

Bruker SMART CCD diffractometer
Absorption correction: multi-scan (*SADABS*; Sheldrick, 1996 ▶) *T*_min_ = 0.943, *T*_max_ = 0.975
10210 measured reflections
6004 independent reflections
5808 reflections with *I* > 2σ(*I*)
*R*_int_ = 0.016

#### Refinement

*R*[*F* ^2^ > 2σ(*F* ^2^)] = 0.042
*wR*(*F* ^2^) = 0.108
*S* = 1.12
6004 reflections
377 parameters
1 restraint
H atoms treated by a mixture of independent and constrained refinement
Δρ_max_ = 0.30 e Å^−3^
Δρ_min_ = −0.25 e Å^−3^
Absolute structure: Flack (1983 ▶), 2440 Friedel pairs
Flack parameter: 0.09 (6)

### 

Data collection: *SMART* (Bruker, 2001 ▶); cell refinement: *SAINT-Plus* (Bruker, 2001 ▶); data reduction: *SAINT-Plus*; program(s) used to solve structure: *SHELXS97* (Sheldrick, 2008 ▶); program(s) used to refine structure: *SHELXL97* (Sheldrick, 2008 ▶); molecular graphics: *PLATON* (Spek, 2009 ▶); software used to prepare material for publication: *SHELXTL* (Sheldrick, 2008 ▶).

## Supplementary Material

Crystal structure: contains datablocks global, I. DOI: 10.1107/S1600536809044596/lh2934sup1.cif

Structure factors: contains datablocks I. DOI: 10.1107/S1600536809044596/lh2934Isup2.hkl

Additional supplementary materials:  crystallographic information; 3D view; checkCIF report

## Figures and Tables

**Table 1:** Hydrogen-bond geometry (Å, °)

*D*—H⋯*A*	*D*—H	H⋯*A*	*D*⋯*A*	*D*—H⋯*A*
N1—H1⋯O4*W* ^i^	1.00 (3)	1.73 (3)	2.689 (3)	159 (2)
N2—H2*A*⋯O1*W*	0.91 (3)	1.83 (3)	2.720 (3)	165 (2)
O1*W*—H1*WA*⋯O2*W*	0.65 (5)	2.07 (5)	2.702 (3)	165 (5)
O1*W*—H1*WB*⋯O1*B* ^ii^	0.74 (4)	2.07 (5)	2.784 (3)	164 (5)
O2*W*—H2*WA*⋯O2*A*	0.74 (5)	2.10 (5)	2.828 (3)	169 (5)
O2*W*—H2*WB*⋯O3*B* ^iii^	0.65 (4)	2.47 (5)	3.075 (3)	156 (7)
O3*W*—H3*WA*⋯O3*A*	0.73 (4)	2.12 (4)	2.844 (3)	173 (5)
O3*W*—H3*WB*⋯O2*B* ^iii^	0.80 (4)	2.07 (4)	2.856 (3)	167 (4)
O4*W*—H4*WA*⋯O1*A*	0.71 (5)	2.14 (5)	2.820 (3)	162 (6)
O4*W*—H4*WB*⋯O3*W* ^iv^	0.86 (5)	1.89 (5)	2.732 (3)	165 (5)
C1*A*—H1*A*1⋯O1*A* ^v^	0.96	2.47	3.117 (3)	125
C1*A*—H1*A*2⋯O1*W* ^i^	0.96	2.54	3.424 (4)	154
C2*A*—H2*A*1⋯O3*A* ^vi^	0.96	2.43	3.382 (4)	170
C2*A*—H2*A*3⋯O2*A* ^vii^	0.96	2.45	3.345 (4)	156
C6*B*—H6*B*⋯O3*B*	0.93	2.49	3.077 (3)	122
C1*B*—H1*B*1⋯O1*B* ^viii^	0.96	2.46	3.253 (3)	140
C1*B*—H1*B*2⋯O1*A* ^ix^	0.96	2.57	3.475 (3)	157
C9*A*—H9*A*⋯O1*A*	0.93	2.40	2.824 (3)	108
C9*B*—H9*B*⋯O1*B*	0.93	2.39	2.815 (3)	108
C2*B*—H2*B*1⋯O2*B* ^x^	0.96	2.36	3.310 (3)	169
C2*B*—H2*B*3⋯O3*B* ^iii^	0.96	2.43	3.286 (3)	149

Symmetry codes: (i) 
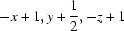
; (ii) 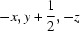; (iii) 
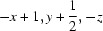
; (iv) 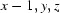; (v) 
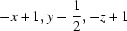
; (vi) 
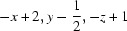
; (vii) 
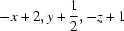
; (viii) 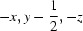; (ix) 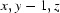; (x) 
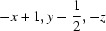
.

**Fig. 1.**
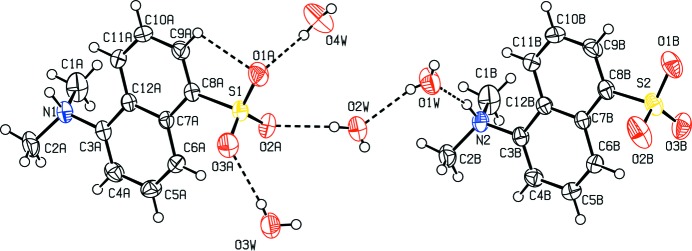
The asymmetric unit of (I), with displacement ellipsoids drawn at the 50% probability level and hydrogen bonds shown as dashed lines.

**Fig. 2.**
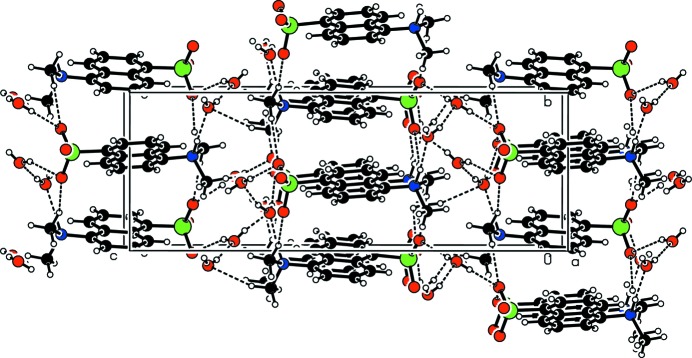
Part of the crystal structure of (I) showing hydrogen bonds as dashed lines.

## supplementary crystallographic information

### Comment

Dansyl acid (5-(dimethylamino)-1-naphthalenesulfonic acid) is an inermidiate that can be used in the preparation of dansyl chloride (Chimiak & Polonski, 1973; Mildenstein, 1971) and is used as a dye due to its good fluorescent property and water solublity. In order to obtain the pure standard sample, the title compound, (I), was crystallized from the technical grade dansyl acid, and we report the crystal stucture herein.

In the molecular structure (Fig. 1), the N atom of the dimethylamino group is protonated. There are two crystallographically independent molecules in the asymmetric unit. All bond lengths and bond angles are as expected. In the crystal structure (Fig.2), the molecules are linked by intermolecular O—H···O, N—H···O and weak C-H···O hydrogen bonds to form a three-dimensional network.

### Experimental

The title compound was crystallized from technical grade dansyl acid. Single crystals suitable for X-ray diffraction were prepared by slow evaporation of a solution of the title compound in water at room temperature.

### Refinement

All H atoms were placed in idealized positions [C—H(methyl)=0.96 Å and C—H(aromatic) =0.93 Å] and included in the refinement in the riding-model approximation, with *U*_iso_(H_methyl_)= 1.5*U*_eq_(C) and *U*_iso_(H_aromatic_) = 1.2*U*_eq_(C). H atoms bonded to N and O atoms were located from the difference maps with the N-H and O-H distances refined freely and U_iso_(H) = 1.2U_eq_(N) and 1.2U_eq_(O).

### Crystal data

**Table:**

C_12_H_13_NO_3_S·2H_2_O	*F*(000) = 608
*M**_r_* = 287.33	*D*_x_ = 1.419 Mg m^−^^3^
Monoclinic, *P*2_1_	Mo *K*α radiation, λ = 0.71073 Å
Hall symbol: P 2yb	Cell parameters from 5479 reflections
*a* = 8.1179 (7) Å	θ = 2.5–28.2°
*b* = 7.7383 (7) Å	µ = 0.26 mm^−^^1^
*c* = 21.4249 (19) Å	*T* = 298 K
β = 91.527 (1)°	Block, colorless
*V* = 1345.4 (2) Å^3^	0.23 × 0.10 × 0.10 mm
*Z* = 4	

### Data collection

**Table:**

Bruker SMART CCD diffractometer	6004 independent reflections
Radiation source: fine-focus sealed tube	5808 reflections with *I* > 2σ(*I*)
graphite	*R*_int_ = 0.016
φ and ω scans	θ_max_ = 28.3°, θ_min_ = 1.9°
Absorption correction: multi-scan (*SADABS*; Sheldrick, 1996)	*h* = −10→10
*T*_min_ = 0.943, *T*_max_ = 0.975	*k* = −10→9
10210 measured reflections	*l* = −28→22

### Refinement

**Table:**

Refinement on *F*^2^	Secondary atom site location: difference Fourier map
Least-squares matrix: full	Hydrogen site location: inferred from neighbouring sites
*R*[*F*^2^ > 2σ(*F*^2^)] = 0.042	H atoms treated by a mixture of independent and constrained refinement
*wR*(*F*^2^) = 0.108	*w* = 1/[σ^2^(*F*_o_^2^) + (0.057*P*)^2^ + 0.1577*P*] where *P* = (*F*_o_^2^ + 2*F*_c_^2^)/3
*S* = 1.12	(Δ/σ)_max_ = 0.001
6004 reflections	Δρ_max_ = 0.30 e Å^−^^3^
377 parameters	Δρ_min_ = −0.25 e Å^−^^3^
1 restraint	Absolute structure: Flack (1983), 2440 Friedel pairs
Primary atom site location: structure-invariant direct methods	Flack parameter: 0.09 (6)

### Special details

**Table:**

Geometry. All e.s.d.'s (except the e.s.d. in the dihedral angle between two l.s. planes) are estimated using the full covariance matrix. The cell e.s.d.'s are taken into account individually in the estimation of e.s.d.'s in distances, angles and torsion angles; correlations between e.s.d.'s in cell parameters are only used when they are defined by crystal symmetry. An approximate (isotropic) treatment of cell e.s.d.'s is used for estimating e.s.d.'s involving l.s. planes.
Refinement. Refinement of *F*^2^ against ALL reflections. The weighted *R*-factor *wR* and goodness of fit *S* are based on *F*^2^, conventional *R*-factors *R* are based on *F*, with *F* set to zero for negative *F*^2^. The threshold expression of *F*^2^ > σ(*F*^2^) is used only for calculating *R*-factors(gt) *etc*. and is not relevant to the choice of reflections for refinement. *R*-factors based on *F*^2^ are statistically about twice as large as those based on *F*, and *R*- factors based on ALL data will be even larger.

### Fractional atomic coordinates and isotropic or equivalent isotropic displacement parameters (Å^2^)

**Table:**

	*x*	*y*	*z*	*U*_iso_*/*U*_eq_	
C1A	0.8222 (4)	0.7568 (4)	0.67513 (13)	0.0591 (7)	
H1A1	0.7141	0.7438	0.6564	0.089*	
H1A2	0.8134	0.7704	0.7195	0.089*	
H1A3	0.8868	0.6561	0.6666	0.089*	
C2A	1.0672 (3)	0.9425 (4)	0.68158 (12)	0.0510 (6)	
H2A1	1.1333	0.8401	0.6787	0.077*	
H2A2	1.0496	0.9686	0.7247	0.077*	
H2A3	1.1230	1.0374	0.6625	0.077*	
C3A	0.9124 (2)	0.8994 (3)	0.57984 (9)	0.0311 (4)	
C4A	1.0527 (3)	0.8477 (3)	0.55268 (11)	0.0387 (5)	
H4A	1.1455	0.8212	0.5772	0.046*	
C5A	1.0577 (3)	0.8341 (3)	0.48737 (11)	0.0392 (5)	
H5A	1.1549	0.8003	0.4688	0.047*	
C6A	0.9221 (2)	0.8697 (3)	0.45084 (10)	0.0342 (4)	
H6A	0.9277	0.8600	0.4077	0.041*	
C7A	0.7725 (2)	0.9213 (3)	0.47795 (9)	0.0279 (4)	
C8A	0.6262 (2)	0.9610 (3)	0.44142 (9)	0.0297 (4)	
C9A	0.4851 (3)	1.0130 (3)	0.46934 (10)	0.0360 (5)	
H9A	0.3914	1.0377	0.4450	0.043*	
C10A	0.4811 (2)	1.0293 (3)	0.53469 (11)	0.0377 (5)	
H10A	0.3849	1.0662	0.5532	0.045*	
C11A	0.6160 (3)	0.9919 (3)	0.57093 (10)	0.0353 (4)	
H11A	0.6107	1.0024	0.6141	0.042*	
C12A	0.7652 (2)	0.9370 (3)	0.54411 (9)	0.0289 (4)	
C1B	0.3679 (4)	−0.0780 (4)	0.17998 (12)	0.0604 (8)	
H1B1	0.2618	−0.1069	0.1621	0.091*	
H1B2	0.3578	−0.0561	0.2238	0.091*	
H1B3	0.4426	−0.1723	0.1741	0.091*	
C2B	0.5991 (3)	0.1247 (4)	0.17579 (10)	0.0431 (5)	
H2B1	0.6733	0.0303	0.1690	0.065*	
H2B2	0.5907	0.1449	0.2198	0.065*	
H2B3	0.6402	0.2268	0.1561	0.065*	
C3B	0.4261 (2)	0.0636 (3)	0.07984 (9)	0.0306 (4)	
C4B	0.5612 (3)	0.0130 (3)	0.04898 (10)	0.0356 (4)	
H4B	0.6583	−0.0142	0.0708	0.043*	
C5B	0.5531 (3)	0.0021 (3)	−0.01663 (11)	0.0391 (5)	
H5B	0.6462	−0.0311	−0.0380	0.047*	
C6B	0.4122 (3)	0.0391 (3)	−0.04930 (10)	0.0339 (4)	
H6B	0.4106	0.0335	−0.0927	0.041*	
C7B	0.2664 (2)	0.0865 (3)	−0.01780 (9)	0.0287 (4)	
C8B	0.1143 (2)	0.1267 (3)	−0.04992 (9)	0.0324 (4)	
C9B	−0.0215 (3)	0.1749 (3)	−0.01754 (11)	0.0403 (5)	
H9B	−0.1195	0.2002	−0.0390	0.048*	
C10B	−0.0137 (3)	0.1863 (3)	0.04774 (12)	0.0442 (6)	
H10B	−0.1067	0.2197	0.0691	0.053*	
C11B	0.1280 (3)	0.1491 (3)	0.08027 (10)	0.0376 (5)	
H11B	0.1306	0.1563	0.1236	0.045*	
C12B	0.2723 (2)	0.0993 (3)	0.04870 (9)	0.0284 (4)	
N1	0.9038 (2)	0.9135 (3)	0.64846 (8)	0.0348 (4)	
H1	0.839 (3)	1.018 (4)	0.6606 (13)	0.042*	
N2	0.4328 (2)	0.0813 (2)	0.14853 (8)	0.0337 (4)	
H2A	0.376 (3)	0.178 (4)	0.1585 (12)	0.040*	
O1A	0.4551 (2)	0.9795 (3)	0.33829 (8)	0.0511 (5)	
O2A	0.6634 (2)	0.7583 (2)	0.34646 (8)	0.0451 (4)	
O3A	0.7434 (2)	1.0568 (3)	0.33579 (8)	0.0504 (5)	
O1B	−0.0802 (2)	0.1413 (3)	−0.14705 (9)	0.0578 (5)	
O2B	0.1916 (3)	0.2632 (3)	−0.15466 (10)	0.0725 (7)	
O3B	0.1561 (2)	−0.0440 (3)	−0.15347 (8)	0.0560 (5)	
O1W	0.3130 (3)	0.3943 (3)	0.18320 (12)	0.0594 (6)	
H1WA	0.366 (5)	0.429 (6)	0.203 (2)	0.089*	
H1WB	0.240 (5)	0.445 (6)	0.173 (2)	0.089*	
O2W	0.5713 (3)	0.5395 (4)	0.24571 (12)	0.0736 (8)	
H2WA	0.589 (6)	0.606 (7)	0.270 (2)	0.110*	
H2WB	0.624 (6)	0.549 (8)	0.223 (2)	0.110*	
O3W	0.9865 (3)	0.9308 (4)	0.25417 (11)	0.0628 (6)	
H3WA	0.920 (5)	0.957 (6)	0.274 (2)	0.094*	
H3WB	0.951 (5)	0.880 (6)	0.224 (2)	0.094*	
O4W	0.2032 (3)	0.7313 (3)	0.32222 (13)	0.0709 (7)	
H4WA	0.279 (5)	0.776 (7)	0.328 (2)	0.106*	
H4WB	0.140 (6)	0.783 (6)	0.295 (2)	0.106*	
S1	0.62169 (6)	0.93747 (7)	0.35870 (2)	0.03361 (13)	
S2	0.09379 (7)	0.11939 (7)	−0.13279 (2)	0.03875 (14)	

### Atomic displacement parameters (Å^2^)

**Table:**

	*U*^11^	*U*^22^	*U*^33^	*U*^12^	*U*^13^	*U*^23^
C1A	0.088 (2)	0.0488 (16)	0.0404 (13)	−0.0201 (15)	−0.0035 (13)	0.0103 (12)
C2A	0.0517 (13)	0.0543 (16)	0.0459 (13)	0.0016 (12)	−0.0224 (10)	−0.0105 (13)
C3A	0.0355 (10)	0.0276 (10)	0.0298 (9)	−0.0024 (7)	−0.0062 (7)	−0.0011 (7)
C4A	0.0331 (10)	0.0371 (12)	0.0454 (12)	0.0024 (9)	−0.0077 (9)	0.0004 (10)
C5A	0.0327 (10)	0.0397 (12)	0.0454 (12)	0.0076 (9)	0.0050 (9)	−0.0031 (10)
C6A	0.0347 (10)	0.0359 (11)	0.0322 (10)	0.0022 (8)	0.0026 (8)	−0.0044 (8)
C7A	0.0302 (8)	0.0250 (9)	0.0284 (9)	−0.0013 (7)	−0.0019 (7)	−0.0036 (8)
C8A	0.0321 (9)	0.0281 (11)	0.0287 (9)	−0.0006 (7)	−0.0039 (7)	−0.0018 (8)
C9A	0.0313 (10)	0.0399 (12)	0.0364 (11)	0.0044 (8)	−0.0056 (8)	−0.0041 (9)
C10A	0.0281 (9)	0.0446 (13)	0.0406 (11)	0.0040 (8)	0.0046 (8)	−0.0095 (10)
C11A	0.0365 (10)	0.0408 (12)	0.0286 (9)	−0.0022 (8)	0.0018 (8)	−0.0085 (9)
C12A	0.0313 (9)	0.0256 (9)	0.0295 (9)	−0.0026 (8)	−0.0018 (7)	−0.0020 (8)
C1B	0.092 (2)	0.0495 (16)	0.0395 (13)	−0.0247 (15)	−0.0014 (13)	0.0128 (12)
C2B	0.0447 (11)	0.0458 (13)	0.0381 (11)	0.0033 (11)	−0.0125 (9)	−0.0074 (11)
C3B	0.0349 (10)	0.0286 (10)	0.0281 (9)	−0.0030 (7)	−0.0031 (7)	0.0017 (7)
C4B	0.0310 (9)	0.0380 (12)	0.0376 (11)	0.0052 (8)	−0.0056 (8)	0.0001 (9)
C5B	0.0333 (10)	0.0448 (13)	0.0393 (11)	0.0032 (9)	0.0043 (8)	−0.0047 (10)
C6B	0.0351 (10)	0.0377 (11)	0.0288 (10)	−0.0002 (8)	0.0007 (8)	−0.0022 (8)
C7B	0.0313 (9)	0.0257 (10)	0.0291 (9)	−0.0014 (7)	−0.0020 (7)	0.0004 (7)
C8B	0.0349 (9)	0.0275 (10)	0.0344 (9)	−0.0016 (8)	−0.0053 (7)	0.0003 (9)
C9B	0.0275 (10)	0.0431 (13)	0.0497 (13)	0.0037 (8)	−0.0083 (9)	−0.0034 (10)
C10B	0.0295 (10)	0.0514 (14)	0.0520 (14)	0.0012 (9)	0.0081 (9)	−0.0140 (11)
C11B	0.0337 (10)	0.0445 (13)	0.0347 (10)	−0.0046 (9)	0.0034 (8)	−0.0077 (10)
C12B	0.0299 (8)	0.0255 (10)	0.0298 (9)	−0.0012 (7)	−0.0013 (7)	0.0007 (7)
N1	0.0424 (9)	0.0313 (10)	0.0302 (8)	−0.0014 (7)	−0.0079 (7)	−0.0011 (7)
N2	0.0409 (9)	0.0335 (10)	0.0265 (8)	−0.0008 (7)	−0.0050 (7)	0.0012 (7)
O1A	0.0496 (9)	0.0619 (12)	0.0411 (9)	0.0064 (8)	−0.0152 (7)	−0.0019 (8)
O2A	0.0619 (10)	0.0390 (9)	0.0343 (8)	0.0010 (8)	−0.0032 (7)	−0.0086 (7)
O3A	0.0625 (11)	0.0479 (11)	0.0408 (9)	−0.0135 (8)	0.0005 (8)	0.0049 (8)
O1B	0.0517 (10)	0.0594 (13)	0.0607 (11)	0.0024 (9)	−0.0275 (8)	−0.0005 (10)
O2B	0.0861 (15)	0.0787 (16)	0.0514 (12)	−0.0402 (13)	−0.0250 (10)	0.0286 (11)
O3B	0.0684 (12)	0.0618 (13)	0.0372 (9)	0.0117 (10)	−0.0103 (8)	−0.0072 (9)
O1W	0.0548 (12)	0.0457 (12)	0.0763 (14)	0.0097 (8)	−0.0231 (10)	−0.0161 (10)
O2W	0.0552 (12)	0.096 (2)	0.0693 (16)	−0.0101 (12)	−0.0061 (10)	−0.0406 (14)
O3W	0.0552 (11)	0.0704 (14)	0.0619 (12)	0.0060 (11)	−0.0140 (9)	−0.0246 (12)
O4W	0.0612 (13)	0.0593 (14)	0.0908 (17)	−0.0175 (10)	−0.0267 (12)	0.0331 (12)
S1	0.0395 (3)	0.0342 (3)	0.0268 (2)	−0.0021 (2)	−0.00519 (18)	−0.0009 (2)
S2	0.0428 (3)	0.0390 (3)	0.0337 (3)	−0.0061 (2)	−0.0122 (2)	0.0076 (2)

### Geometric parameters (Å, °)

**Table:**

C1A—N1	1.502 (3)	C3B—C4B	1.354 (3)
C1A—H1A1	0.9600	C3B—C12B	1.427 (3)
C1A—H1A2	0.9600	C3B—N2	1.477 (3)
C1A—H1A3	0.9600	C4B—C5B	1.408 (3)
C2A—N1	1.505 (3)	C4B—H4B	0.9300
C2A—H2A1	0.9600	C5B—C6B	1.356 (3)
C2A—H2A2	0.9600	C5B—H5B	0.9300
C2A—H2A3	0.9600	C6B—C7B	1.426 (3)
C3A—C4A	1.353 (3)	C6B—H6B	0.9300
C3A—C12A	1.431 (3)	C7B—C12B	1.428 (3)
C3A—N1	1.478 (3)	C7B—C8B	1.432 (3)
C4A—C5A	1.405 (3)	C8B—C9B	1.369 (3)
C4A—H4A	0.9300	C8B—S2	1.780 (2)
C5A—C6A	1.362 (3)	C9B—C10B	1.401 (3)
C5A—H5A	0.9300	C9B—H9B	0.9300
C6A—C7A	1.418 (3)	C10B—C11B	1.360 (3)
C6A—H6A	0.9300	C10B—H10B	0.9300
C7A—C12A	1.425 (3)	C11B—C12B	1.421 (3)
C7A—C8A	1.438 (3)	C11B—H11B	0.9300
C8A—C9A	1.367 (3)	N1—H1	1.00 (3)
C8A—S1	1.781 (2)	N2—H2A	0.91 (3)
C9A—C10A	1.407 (3)	O1A—S1	1.4474 (17)
C9A—H9A	0.9300	O2A—S1	1.4525 (18)
C10A—C11A	1.357 (3)	O3A—S1	1.4481 (18)
C10A—H10A	0.9300	O1B—S2	1.4473 (17)
C11A—C12A	1.419 (3)	O2B—S2	1.452 (2)
C11A—H11A	0.9300	O3B—S2	1.436 (2)
C1B—N2	1.507 (3)	O1W—H1WA	0.65 (5)
C1B—H1B1	0.9600	O1W—H1WB	0.74 (4)
C1B—H1B2	0.9600	O2W—H2WA	0.74 (5)
C1B—H1B3	0.9600	O2W—H2WB	0.65 (4)
C2B—N2	1.495 (3)	O3W—H3WA	0.73 (4)
C2B—H2B1	0.9600	O3W—H3WB	0.80 (4)
C2B—H2B2	0.9600	O4W—H4WA	0.71 (5)
C2B—H2B3	0.9600	O4W—H4WB	0.86 (5)
			
N1—C1A—H1A1	109.5	C12B—C3B—N2	117.15 (17)
N1—C1A—H1A2	109.5	C3B—C4B—C5B	119.20 (19)
H1A1—C1A—H1A2	109.5	C3B—C4B—H4B	120.4
N1—C1A—H1A3	109.5	C5B—C4B—H4B	120.4
H1A1—C1A—H1A3	109.5	C6B—C5B—C4B	121.30 (19)
H1A2—C1A—H1A3	109.5	C6B—C5B—H5B	119.3
N1—C2A—H2A1	109.5	C4B—C5B—H5B	119.3
N1—C2A—H2A2	109.5	C5B—C6B—C7B	120.63 (19)
H2A1—C2A—H2A2	109.5	C5B—C6B—H6B	119.7
N1—C2A—H2A3	109.5	C7B—C6B—H6B	119.7
H2A1—C2A—H2A3	109.5	C6B—C7B—C12B	118.94 (17)
H2A2—C2A—H2A3	109.5	C6B—C7B—C8B	122.98 (18)
C4A—C3A—C12A	122.02 (19)	C12B—C7B—C8B	118.05 (16)
C4A—C3A—N1	120.80 (18)	C9B—C8B—C7B	120.73 (19)
C12A—C3A—N1	117.15 (17)	C9B—C8B—S2	117.31 (16)
C3A—C4A—C5A	119.84 (19)	C7B—C8B—S2	121.95 (15)
C3A—C4A—H4A	120.1	C8B—C9B—C10B	120.50 (19)
C5A—C4A—H4A	120.1	C8B—C9B—H9B	119.8
C6A—C5A—C4A	120.8 (2)	C10B—C9B—H9B	119.8
C6A—C5A—H5A	119.6	C11B—C10B—C9B	120.9 (2)
C4A—C5A—H5A	119.6	C11B—C10B—H10B	119.6
C5A—C6A—C7A	120.7 (2)	C9B—C10B—H10B	119.6
C5A—C6A—H6A	119.7	C10B—C11B—C12B	120.7 (2)
C7A—C6A—H6A	119.7	C10B—C11B—H11B	119.7
C6A—C7A—C12A	119.34 (17)	C12B—C11B—H11B	119.7
C6A—C7A—C8A	122.78 (17)	C11B—C12B—C3B	123.50 (18)
C12A—C7A—C8A	117.89 (16)	C11B—C12B—C7B	119.17 (17)
C9A—C8A—C7A	120.95 (18)	C3B—C12B—C7B	117.32 (17)
C9A—C8A—S1	118.06 (15)	C3A—N1—C1A	110.61 (17)
C7A—C8A—S1	120.97 (14)	C3A—N1—C2A	114.57 (18)
C8A—C9A—C10A	120.24 (19)	C1A—N1—C2A	109.5 (2)
C8A—C9A—H9A	119.9	C3A—N1—H1	110.9 (17)
C10A—C9A—H9A	119.9	C1A—N1—H1	108.1 (16)
C11A—C10A—C9A	120.70 (19)	C2A—N1—H1	102.7 (16)
C11A—C10A—H10A	119.6	C3B—N2—C2B	114.75 (16)
C9A—C10A—H10A	119.6	C3B—N2—C1B	111.46 (17)
C10A—C11A—C12A	121.12 (19)	C2B—N2—C1B	109.40 (19)
C10A—C11A—H11A	119.4	C3B—N2—H2A	107.8 (17)
C12A—C11A—H11A	119.4	C2B—N2—H2A	100.6 (17)
C11A—C12A—C7A	119.11 (17)	C1B—N2—H2A	112.4 (17)
C11A—C12A—C3A	123.59 (18)	H1WA—O1W—H1WB	119 (5)
C7A—C12A—C3A	117.29 (17)	H2WA—O2W—H2WB	109 (6)
N2—C1B—H1B1	109.5	H3WA—O3W—H3WB	111 (4)
N2—C1B—H1B2	109.5	H4WA—O4W—H4WB	112 (5)
H1B1—C1B—H1B2	109.5	O1A—S1—O3A	113.26 (12)
N2—C1B—H1B3	109.5	O1A—S1—O2A	112.35 (11)
H1B1—C1B—H1B3	109.5	O3A—S1—O2A	112.54 (11)
H1B2—C1B—H1B3	109.5	O1A—S1—C8A	105.78 (10)
N2—C2B—H2B1	109.5	O3A—S1—C8A	106.03 (10)
N2—C2B—H2B2	109.5	O2A—S1—C8A	106.16 (10)
H2B1—C2B—H2B2	109.5	O3B—S2—O1B	112.84 (12)
N2—C2B—H2B3	109.5	O3B—S2—O2B	112.01 (14)
H2B1—C2B—H2B3	109.5	O1B—S2—O2B	112.41 (14)
H2B2—C2B—H2B3	109.5	O3B—S2—C8B	108.15 (11)
C4B—C3B—C12B	122.54 (18)	O1B—S2—C8B	105.76 (10)
C4B—C3B—N2	120.31 (18)	O2B—S2—C8B	105.06 (11)
			
C12A—C3A—C4A—C5A	1.7 (3)	C7B—C8B—C9B—C10B	0.2 (4)
N1—C3A—C4A—C5A	179.6 (2)	S2—C8B—C9B—C10B	−178.97 (19)
C3A—C4A—C5A—C6A	−1.0 (4)	C8B—C9B—C10B—C11B	−0.3 (4)
C4A—C5A—C6A—C7A	0.0 (4)	C9B—C10B—C11B—C12B	0.6 (4)
C5A—C6A—C7A—C12A	0.5 (3)	C10B—C11B—C12B—C3B	178.0 (2)
C5A—C6A—C7A—C8A	179.8 (2)	C10B—C11B—C12B—C7B	−0.8 (3)
C6A—C7A—C8A—C9A	−178.9 (2)	C4B—C3B—C12B—C11B	178.6 (2)
C12A—C7A—C8A—C9A	0.4 (3)	N2—C3B—C12B—C11B	−0.4 (3)
C6A—C7A—C8A—S1	3.0 (3)	C4B—C3B—C12B—C7B	−2.5 (3)
C12A—C7A—C8A—S1	−177.72 (15)	N2—C3B—C12B—C7B	178.49 (17)
C7A—C8A—C9A—C10A	0.3 (3)	C6B—C7B—C12B—C11B	179.1 (2)
S1—C8A—C9A—C10A	178.41 (18)	C8B—C7B—C12B—C11B	0.7 (3)
C8A—C9A—C10A—C11A	−0.8 (4)	C6B—C7B—C12B—C3B	0.1 (3)
C9A—C10A—C11A—C12A	0.6 (4)	C8B—C7B—C12B—C3B	−178.23 (19)
C10A—C11A—C12A—C7A	0.0 (3)	C4A—C3A—N1—C1A	−99.3 (3)
C10A—C11A—C12A—C3A	178.6 (2)	C12A—C3A—N1—C1A	78.7 (3)
C6A—C7A—C12A—C11A	178.8 (2)	C4A—C3A—N1—C2A	25.0 (3)
C8A—C7A—C12A—C11A	−0.5 (3)	C12A—C3A—N1—C2A	−157.0 (2)
C6A—C7A—C12A—C3A	0.2 (3)	C4B—C3B—N2—C2B	28.3 (3)
C8A—C7A—C12A—C3A	−179.17 (19)	C12B—C3B—N2—C2B	−152.7 (2)
C4A—C3A—C12A—C11A	−179.8 (2)	C4B—C3B—N2—C1B	−96.8 (3)
N1—C3A—C12A—C11A	2.2 (3)	C12B—C3B—N2—C1B	82.2 (3)
C4A—C3A—C12A—C7A	−1.2 (3)	C9A—C8A—S1—O1A	−1.0 (2)
N1—C3A—C12A—C7A	−179.19 (18)	C7A—C8A—S1—O1A	177.14 (17)
C12B—C3B—C4B—C5B	2.9 (3)	C9A—C8A—S1—O3A	119.53 (19)
N2—C3B—C4B—C5B	−178.1 (2)	C7A—C8A—S1—O3A	−62.32 (19)
C3B—C4B—C5B—C6B	−0.9 (4)	C9A—C8A—S1—O2A	−120.57 (18)
C4B—C5B—C6B—C7B	−1.5 (4)	C7A—C8A—S1—O2A	57.58 (19)
C5B—C6B—C7B—C12B	1.8 (3)	C9B—C8B—S2—O3B	−130.4 (2)
C5B—C6B—C7B—C8B	−179.9 (2)	C7B—C8B—S2—O3B	50.4 (2)
C6B—C7B—C8B—C9B	−178.7 (2)	C9B—C8B—S2—O1B	−9.3 (2)
C12B—C7B—C8B—C9B	−0.4 (3)	C7B—C8B—S2—O1B	171.49 (18)
C6B—C7B—C8B—S2	0.4 (3)	C9B—C8B—S2—O2B	109.8 (2)
C12B—C7B—C8B—S2	178.75 (15)	C7B—C8B—S2—O2B	−69.4 (2)

### Hydrogen-bond geometry (Å, °)

**Table:**

*D*—H···*A*	*D*—H	H···*A*	*D*···*A*	*D*—H···*A*
N1—H1···O4W^i^	1.00 (3)	1.73 (3)	2.689 (3)	159 (2)
N2—H2A···O1W	0.91 (3)	1.83 (3)	2.720 (3)	165 (2)
O1W—H1WA···O2W	0.65 (5)	2.07 (5)	2.702 (3)	165 (5)
O1W—H1WB···O1B^ii^	0.74 (4)	2.07 (5)	2.784 (3)	164 (5)
O2W—H2WA···O2A	0.74 (5)	2.10 (5)	2.828 (3)	169 (5)
O2W—H2WB···O3B^iii^	0.65 (4)	2.47 (5)	3.075 (3)	156 (7)
O3W—H3WA···O3A	0.73 (4)	2.12 (4)	2.844 (3)	173 (5)
O3W—H3WB···O2B^iii^	0.80 (4)	2.07 (4)	2.856 (3)	167 (4)
O4W—H4WA···O1A	0.71 (5)	2.14 (5)	2.820 (3)	162 (6)
O4W—H4WB···O3W^iv^	0.86 (5)	1.89 (5)	2.732 (3)	165 (5)
C1A—H1A1···O1A^v^	0.96	2.47	3.117 (3)	125
C1A—H1A2···O1W^i^	0.96	2.54	3.424 (4)	154
C2A—H2A1···O3A^vi^	0.96	2.43	3.382 (4)	170
C2A—H2A3···O2A^vii^	0.96	2.45	3.345 (4)	156
C6B—H6B···O3B	0.93	2.49	3.077 (3)	122
C1B—H1B1···O1B^viii^	0.96	2.46	3.253 (3)	140
C1B—H1B2···O1A^ix^	0.96	2.57	3.475 (3)	157
C9A—H9A···O1A	0.93	2.40	2.824 (3)	108
C9B—H9B···O1B	0.93	2.39	2.815 (3)	108
C2B—H2B1···O2B^x^	0.96	2.36	3.310 (3)	169
C11A—H11A···N1	0.93	2.57	2.894 (3)	101
C11B—H11B···N2	0.93	2.56	2.888 (3)	101
C2B—H2B3···O3B^iii^	0.96	2.43	3.286 (3)	149

Symmetry codes: (i) −*x*+1, *y*+1/2, −*z*+1; (ii) −*x*, *y*+1/2, −*z*; (iii) −*x*+1, *y*+1/2, −*z*; (iv) *x*−1, *y*, *z*; (v) −*x*+1, *y*−1/2, −*z*+1; (vi) −*x*+2, *y*−1/2, −*z*+1; (vii) −*x*+2, *y*+1/2, −*z*+1; (viii) −*x*, *y*−1/2, −*z*; (ix) *x*, *y*−1, *z*; (x) −*x*+1, *y*−1/2, −*z*.
